# Experimental Inoculation of Juvenile Rhesus Macaques with Primate Enteric Caliciviruses

**DOI:** 10.1371/journal.pone.0037973

**Published:** 2012-05-30

**Authors:** Karol Sestak, Stephanie Feely, Brittney Fey, Jason Dufour, Edwin Hargitt, Xavier Alvarez, Bapi Pahar, Nicole Gregoricus, Jan Vinjé, Tibor Farkas

**Affiliations:** 1 Tulane National Primate Research Center, Covington, Louisiana, United States of America; 2 Tulane University School of Medicine, Covington, Louisiana, United States of America; 3 Cincinnati Children's Hospital Medical Center, Cincinnati, Ohio, United States of America; 4 University of Cincinnati College of Medicine, Cincinnati, Ohio, United States of America; 5 Centers for Disease Control and Prevention, Atlanta, Georgia, United States of America; University of Ottawa, Canada

## Abstract

**Background:**

Tissue culture-adapted Tulane virus (TV), a GI.1 rhesus enteric calicivirus (ReCV), and a mixture of GII.2 and GII.4 human norovirus (NoV)-containing stool sample were used to intrastomacheally inoculate juvenile rhesus macaques (*Macaca mulatta*) in order to evaluate infection caused by these viruses.

**Methodology & Findings:**

Two of the three TV-inoculated macaques developed diarrhea, fever, virus-shedding in stools, inflammation of duodenum and 16-fold increase of TV-neutralizing (VN) serum antibodies but no vomiting or viremia. No VN-antibody responses could be detected against a GI.2 ReCV strain FT285, suggesting that TV and FT285 represent different ReCV serotypes. Both NoV-inoculated macaques remained asymptomatic but with demonstrable virus shedding in one animal. Examination of duodenum biopsies of the TV-inoculated macaques showed lymphocytic infiltration of the lamina propria and villous blunting. TV antigen-positive (TV+) cells were detected in the lamina propria. In most of the TV+ cells TV co-localized perinuclearly with calnexin – an endoplasmic reticulum protein. A few CD20+TV+ double-positive B cells were also identified in duodenum. To corroborate the authenticity of CD20+TV+ B cells, *in vitro* cultures of peripheral blood mononuclear cells (PBMCs) from healthy macaques were inoculated with TV. Multicolor flow cytometry confirmed the presence of TV antigen-containing B cells of predominantly CD20+HLA-DR+ phenotype. A 2-log increase of viral RNA by 6 days post inoculation (p<0.05) suggested active TV replication in cultured lymphocytes.

**Conclusions/Significance:**

Taken together, our results show that ReCVs represent an alternative cell culture and animal model to study enteric calicivirus replication, pathogenesis and immunity.

## Introduction

Caliciviruses (CV) are small, non-enveloped, icosahedral viruses with a ∼7.5–8.5 kb positive sense, single stranded, polyadenylated RNA genome. The *Caliciviridae* family consists of five established genera, *Norovirus, Sapovirus, Lagovirus, Vesivirus* and *Nebovirus*. Rhesus enteric CVs (*Recovirus*), St. Valerian-like viruses (*Valovirus*) and chicken CVs represent three additional, yet unassigned CV genera [1]–[3]. Noroviruses (NoV) are important etiologic agents of acute gastroenteritis in humans and account for the majority of nonbacterial gastroenteritis outbreaks as well as >50% of all food-related gastroenteritis outbreaks [4], [5]. NoVs can be subdivided into five genogroups (GI-V) and at least 33 genotypes [6], [7]. No robust cell culture or *in vivo* model exists to study human NoVs.

The first attempt to develop a primate model for human NoV gastroenteritis dates back to 1978 when Wyatt and colleagues induced asymptomatic infection of chimpanzees by the Norwalk virus [8]. Since then, further efforts have been made with porcine, bovine, murine and other animal hosts including non-human primate (NHP) species to establish a model that would resemble human NoV gastroenteritis [9]–[26]. Despite that substantial achievements have been made with these models, there is still a demand for another alternative that would simultaneously address human NoV-induced clinical illness as well as reflect biological features of human NoVs including their genetic, antigenic and HBGA diversity while being utilized *in vivo* and *in vitro*.

Our group recently discovered and characterized a new group of enteric CVs of rhesus monkey host origin with the proposed name Recoviruses (ReCVs) [1], [27], [28]. Enteric CVs frequently infect colonies with captive NHPs [1], [19], [27], [29], [30]. These reports are based on detection of serum antibodies, electron microscopy or presence of viral RNA in stool samples. The extent of ReCV seroprevalence measured by VN antibodies (as high as 80%) in NHPs colonies is conditioned by host species, age, type of housing and colony [Sestak and Farkas, unpublished]. It is not clear, however, if reactivity of NHP serum samples with human NoVs reflects CV interspecies transmission or cross-reactivity between CVs of different primate hosts [19], [27]. Studies conducted with human NoV-inoculated NHPs suggest that there are differences in susceptibility to infection not only among different host species but also within species – likely caused by individual genetic resistance factors although possible differences in adaptive immunity play an important role as well. While some primates remain asymptomatic after infection, others exhibit diarrhea, vomiting, and dehydration, resembling the clinical course of human NoV gastroenteritis [8], [10], [12], [31]. Robust, virus-specific seroconversion and virus shedding in stools up to three weeks post inoculation have only been reproduced in NHPs. In order to induce symptomatic infection, serological and HBGA pre-screening of candidate animals need to be considered prior to experimental challenge. The main objective of this study was to explore the suitability of juvenile rhesus macaques as a TV infection model to study enteric calicivirus replication, pathogenesis and immunity.

## Results

### Experimental inoculation of juvenile rhesus macaques with TV induces clinical symptoms of disease

**Figure 1:**
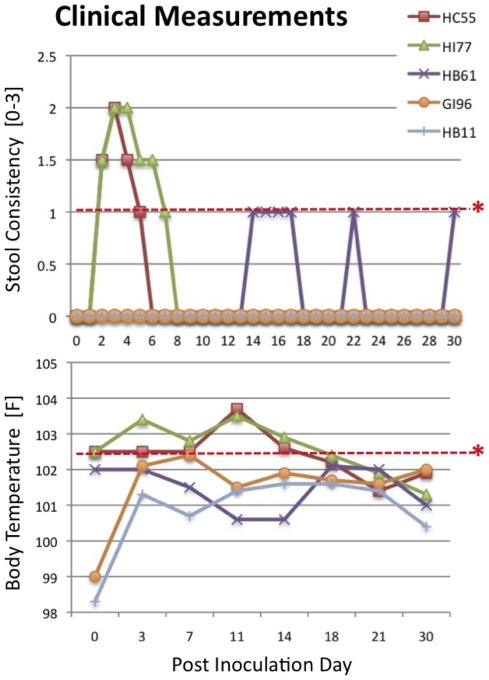
Stool consistency and body temperature measurements. Stool consistency measurements indicate onset of diarrhea (>1.0 red line cut-off) upon inoculation. Two of the three TV-inoculated macaques (HC55 and HI77) showed symptoms of diarrhea up to post-inoculation day (PID) 6 and 8. The two human NoV-inoculated animals (GI96 and HB11) did not show diarrhea. HC55 and HI77 macaques developed elevated body temperature (>102.5 F; red line cut-off) during the first two weeks following TV inoculation, while body temperatures of the other animals remained normal. Definition of diarrhea scale: (0 = normal/dry, 0.5 = normal, 1.0 = normal/soft, 1.5 = pasty, 2.0 = semiliquid, 2.5 = watery), and fever: (<102.5 = normal, 102.5 = borderline, >102.5 = fever).

**Figure 2:**
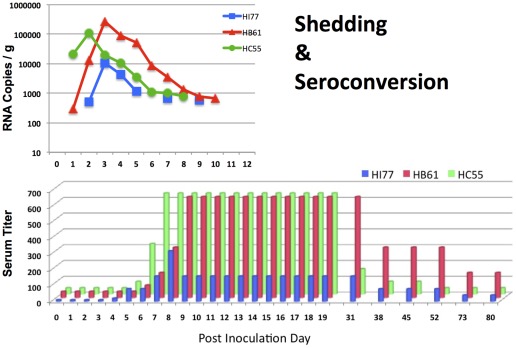
Virus shedding in stools and virus-neutralizing (VN) serum antibody response following TV inoculation. All of the three TV-inoculated macaques showed presence of virus-specific RNA in stools (upper panel) and developed VN antibody responses (≥4-fold increase) by PID 7 (lower panel). Duration of shedding ranged in TV-inoculated macaques between 8–10 days while reaching the peak of ∼10^5^ RNA copies per gram of stools in HB61 and HC55 and ∼10^4^ in HI77. The detection limit of the assay was 10–100 copies per reaction. Antibody serum levels reached their peak in all three macaques by PID 7–9 (1∶160–1∶640) while returned to pre-inoculation levels by PID 80.

Intragastric inoculation of TV of two of the three juvenile macaques (HC55 and HI77) resulted in diarrhea and fever within two days after virus inoculation (Fig. 1) whereas no clinical symptoms were observed for HB61 despite that all three macaques shed TV. The presence of viral genome was measured in cell-free plasma by qRT-PCR. No vomiting and viremia was detected in any of the animals. All three TV-inoculated animals shed the virus for at least 8 days with peak shedding levels of >10^5^ TV-RNA copies per gram of stools in HB61 and HC55 and ≥10^4^ TV-RNA copies per gram of stools in HI77 (Fig. 2). Considering that juvenile macaques produce on average 80±20 grams of stool daily, the amount of virus shed in HC55 and HB61 macaques exceeded the amount received via inoculum (Tables 1 and 2).

**Table 1:** Rhesus macaques and enteric caliciviruses used for experimental inoculation.

	Rhesus	Age		Virus Inoculum, Genotype	Virus Stock	Dose per Inoculation
N	Macaque	[Years]	Subspecies	& Host of Origin	Designation	[RNA copies per animal]
1	HC55	2.5	Indian	TV, GI.1, rhesus^a^	M33 3/2	10 ml (1×0^6^)
2	HI77	2.5	Indian	TV, GI.1, rhesus	M33 3/2	10 ml (1×10^6^)
3	HB61	2.5	Indian	TV, GI.1, rhesus	M33 3/2	10 ml (1×10^6^)
4	HB11	3.0	Indian	NoV, GII/2 & GII/4, human^b^	NF2002, 2003	3 ml (1×10^5^)^c^
5	GI96	4.0	Indian	NoV, GII/2 & GII/4, human	NF2002, 2003	3 ml (1×10^5^)

a Tulane Virus i.e. rhesus enteric CV; ^b^Human NoVs GenBank #s: JQ320072 and JQ320073; ^c^Bacteria-free filtrate of 20% stool suspension in PBS. TV and NoV-inoculated animals were kept separated in devoted BSL2 rooms. The total dosage of TV was 10^6^ viral RNA copies per animal.

**Table 2:** Virus shedding in stools following experimental inoculation.

	Rhesus	Virus	Virus	Duration of	Maximum Shedding
N	Macaque	Inoculum	Shed in Stools	Shedding [Days]	[RNA copies per gram of stools / day]
1	HC55	TV (GI.1)	TV (GI.1)	8	113,000 (day 2)
2	HI77	TV (GI.1)	TV (GI.1)	9	10,600 (day 3)
3	HB61	TV (GI.1)	TV (GI.1)	10	273,300 (day 3)
4	HB11	NoV (GII.2 & GII.4)	NoV (GII.2 & GII.4)	13	76,400 (day 9)
5	GI96	NoV (GII.2 & GII.4)	NoV (GII.2)	4	NA^a^

Virus shedding for the TV-inoculated animals started on day 1 (HC55 and HI77) and on day 2 (HB61). For the NoV-inoculated animals virus shedding started on day 1 (HB11) and day 2 (GI96) as determined by nested RT-PCR. No samples from GI96 were however positive by qRT-PCR and only samples corresponding to HB11 days 1, 2 and 9 were positive and quantitatively evaluated. Considering that juvenile rhesus macaques of 2.5–4.0 years of age produce daily 80±20 grams of stool, the three TV-inoculated and HB11 macaques produced more virus in stools than what they received in inoculum.^ a^not applicable.

No clinical symptoms of diarrhea and fever were observed in the two NoV-inoculated macaques although GI96 met at one time point the definition of “borderline” fever (102.5 F). Viral RNA was detected in fecal specimens from macaque HB11 up to PID 13 and macaque GI96 up to PID 4 by strain-specific nested RT-PCR (Table 2). Both GII.2 and GII.4 viruses were shed in macaque HB11 while only GII.2 virus was detected in macaque GI96. Likely because a lower sensitivity of the quantitative real-time RT-PCR (20–200 RNA copies for GII strains per reaction) compared to nested RT-PCR, only PID 1, 2 and 9 samples from HB11 but none of the samples from GI96 could be evaluated quantitatively (Table 2). Considering that juvenile rhesus macaques of 2.5–4.0 years of age produce daily 80±20 grams of stool, the HB11 macaque produced more virus in stools than what was received in inoculum.

### Serum antibody responses to TV

All three TV-inoculated macaques developed a VN serum antibody response (≥4-fold increase) by PID 7 (Fig. 2). VN antibodies reached their peak at PID 7–9 (1∶160–1∶640), persisted at their peak levels up to PID 30–38 after which they gradually started to decline (Fig. 2). By PID 80, VN antibody levels in all three TV-inoculated animals dropped to almost pre-inoculation levels. None of the TV (GI.1 genotype)-inoculated animals developed VN antibody response against FT285–a GI.2 ReCV. A rise in NoV-specific antibody responses against Minerva (GII.4) VLPs could not be detected in the NoV-inoculated macaques (HB11 and GI96) which had consistent anti-Minerva serum ELISA antibody titers of 1∶50 and 1∶800 throughout the experiment, respectively.

### Histopathological changes in small intestine

**Figure 3:**
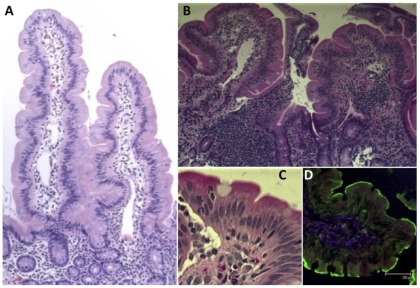
Duodenum biopsies from healthy control and TV-inoculated macaques. A– Duodenum tissue from healthy control macaque with normal tissue architecture of intestinal villi. Magnification 10x. B– Duodenum tissue obtained at PID 3 from TV-inoculated HC55 macaque: Superficial mucosa contains diffuse mononuclear cell infiltrate with formation of an intra-epithelial lymphoid follicle. Villi are slightly blunted. Magnification 10x. C– Superficial mucosa of TV-inoculated macaque contains macrophages, plasma cells and scattered eosinophils. Magnification 40x. D– The brush border (green lining = villi) was preserved in TV-inoculated macaques. Purple blue cells indicate underlying subepithelium (TG2 is a digestive enzyme).

H&E-stained duodenum tissues from TV- and NoV-inoculated macaques were compared with tissues from normal, healthy, age-matched controls (Fig. 3). While no significant damage was seen in the intestinal epithelium, in both TV- and NoV-inoculated animals, mononuclear, lymphocytic infiltration was observed in the lamina propria. In addition, moderate blunting and distention of the villi was observed in all three TV-inoculated animals (Fig. 3). No histopathological lesions were observed in control macaque intestinal tissues.

### Visualization of TV in small intestinal biopsies

**Figure 4:**
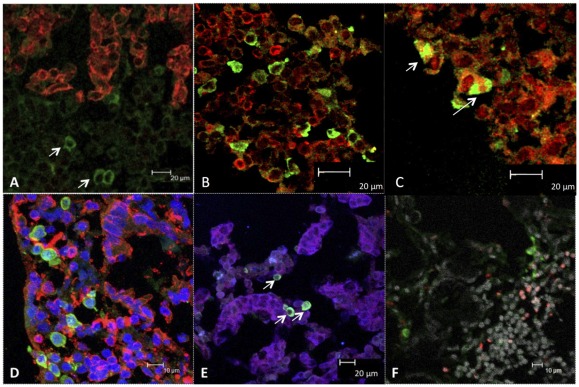
Immunofluorescent confocal microscopy of duodenum biopsies from TV-inoculated macaque HC55. A– The cytokeratin+ epithelial cells (red) did not co-localize with TV (green). The TV+ cells appear in subepithelium i.e. lamina propria (arrows). B– The subepithelial CD3+ T lymphocytes (red) did not co-localize with TV (green). C– Co-localization of some of the CD20+ B cells (red) with TV (green) is indicated by arrows. D– The IBA-1+ macrophages (red) did not co-localize with TV (green). Nuclear DNA is in blue. E– The CD11c+ myeloid dendritic cells (red) did not co-localize with TV (green) while co-localization between CD11c+ and calnexin (blue) markers resulted in purple cell coloration. The co-localization of TV+ cells with calnexin is indicated by arrows. F– Absence of co-localization between TV (green) and TUNEL (red) markers indicates that TV-infected cells did not undergo apoptosis. Differential interference contrast (gray) is highlighted for the better visualization of tissue architecture.

**Figure 5:**
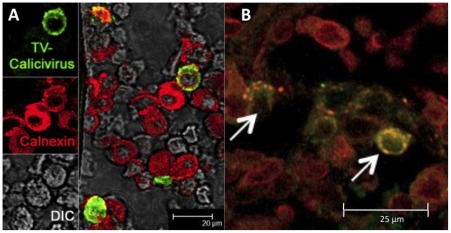
Colocalization of TV and calnexin antigens inside the HC55 macaque's duodenum lamina propria. A– While most of the cells are calnexin-single-positive, simultaneous presence of both TV (green) and calnexin (red) antigens is seen in some of the (yellowish) cells. DIC– differential interference contrast. B– Spectral overlap of TV and calnexin antigens is shown by arrows.

In order to visualize and to characterize TV-infected cells directly in small intestine, duodenum biopsies were collected at PID 3 and studied by confocal microscopy. Antibodies specific to human T-, B-, epithelial, dendritic, apoptotic cells as well as macrophages and endoplasmic protein calnexin were used in conjunction with TV antibodies (Figs. 4 and 5). Consistent with the histopathological findings, no TV antigen was detected in epithelial cells. Nevertheless, numerous TV+ cells were observed within the lamina propria. No TV+ cells were detected in biopsies from uninfected control macaques (Fig. S1). Many of the TV antigens exhibited perinuclear fluorescence while TV-specific antibodies colocalized with calnexin in some cells (Fig. 5; Fig. S2). Some of the TV+ cells also stained with CD20, a B cell-specific marker (Fig. 4C).

### Quantitative analysis of *in vitro* TV-inoculated PBMCs

**Figure 6:**
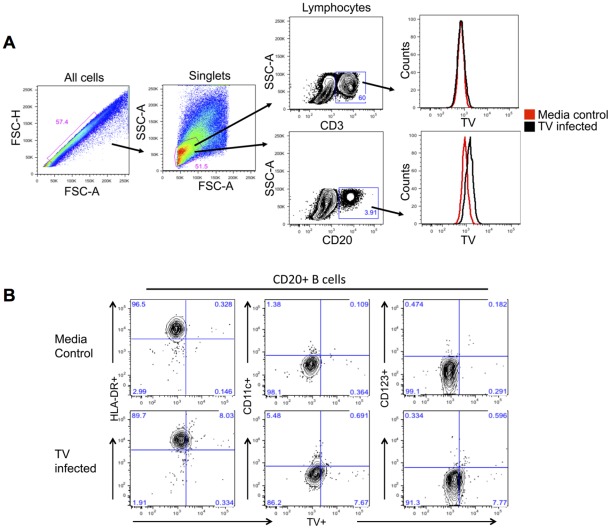
Flow cytometry detection of TV antigens-containing cells *in vitro*. A– PBMCs isolated from healthy rhesus macaques were used for *in vitro* inoculation with TV. After being cultured *in vitro* for 6 days, cells were sorted out into CD3+ T cell and CD20+ B cell populations. Presence of TV antigens was detected in CD20+ B cells as shown by histograms (black peak). B– The CD20+ B cells were further subdivided into populations expressing the HLA-DR, CD11c or CD123 antigens. Presence of TV was revealed predominantly in CD20+HLA-DR+ cells while some of the other lymphocyte populations including the CD20+CD11c+ and CD20+CD123+ B cells also contained TV antigens.

**Figure 7:**
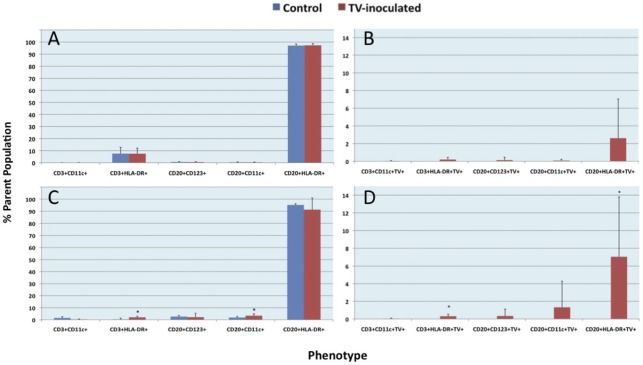
TV-antigen containing lymphocyte populations in cultured, TV-inoculated PBMCs. A & B– One day-old cultures of TV-inoculated PBMCs show the trend (p>0.05) for TV antigen expression by CD20+HLA-DR+ B cells. C & D– Six days-old cultures show significant presence of TV antigens predominantly inside the CD20+HLA-DR+ cells (p<0.05). Several other T and B cell subsets also contain TV while no virus was detected in negative control, mock-inoculated PBMCs. Asterisks indicate significant differences between the TV-inoculated and control cells.

Because suitability of intestinal endoscopic (pin-head size) biopsies for quantitative cell phenotypic analysis by confocal microscopy is limited, *in vitro* experiments with TV-inoculated PBMCs obtained from eight healthy macaques were performed to corroborate the presence of CD20+TV+ B cells in the biopsy samples and to assess the susceptibility of mononuclear cell populations such as T and B lymphocytes to TV infection. *In vitro* cultures of PBMCs from 8 healthy rhesus macaques were used. TV and negative control media-inoculated cells were harvested at 2 h, 24 h and 6 days PI and tested by multicolor flow cytometry and/or qPCR to determine the number of TV+ cells and virus load. Two populations of interest were identified by flow cytometry: CD3+ T and CD20+ B cells (Fig. 6A). Based on distinct subset of CD20+TV+ cells identified within CD20+ B cell population (Fig. 6A), presented data focus on B cells while T cell populations and negative controls are shown for comparison (Fig. 6B, Fig. 7). The two “parent” (CD20+ and CD3+) populations were further sub-divided into CD20+HLA-DR+, CD20+CD11c+, CD20+CD123+, CD3+HLA-DR+ and CD3+CD11c+ cells, corresponding to five different B and T cell subsets, to determine which of these cells, if any, contained the TV. The distribution of five of the above lymphocyte subsets was similar between TV-inoculated and media control PBMCs, as well as between cultures harvested at 24 h and 6 days PI, with CD20+HLA-DR+ B cell subset being the dominant (Fig. 7A, C). In both CD3+ and CD20+ cell populations HLA-DR+ cells were the most abundant, representing over 90% of cells while CD11c+ and/or CD123+ cells represented less than 10%. All of the five lymphocyte subsets contained TV+ cells, but only the CD20+HLA-DR+TV+ cells surpassed 2% (p>0.05) of the parent (CD20+HLA-DR+) population at 24 h PI (Fig. 7B). Moreover, the proportion of CD20+HLA-DR+TV+ cells increased to >6% (p<0.05) of the parent population by PID 6 (Fig. 7D). Such an increased proportion of TV+ cells in cultured PBMCs was corroborated by qPCR. A significant increase in virus load was detected within 1–6 days PI while no virus was detected in non-inoculated controls: The average viral RNA copy number per well by PID 1 (1.3×10^6^) and PID 6 (2.8×10^6^) was higher (p<0.05) than the RNA copy number measured by 2 hours PI (2.3×10^5^).

## Discussion

Human NoVs are a common cause of epidemic and sporadic acute gastroenteritis worldwide [4], [32]. The prototype Norwalk virus was discovered in 1972 [33], however NoV research and the development of prevention strategies have since been hampered by the lack of robust cell culture system and animal model that closely mimics the clinical symptoms of NoV gastroenteritis in humans. Despite these difficulties, NoV VLP vaccines are in early phase clinical trials [34], [35]. Evaluation of these vaccines largely depends on challenge studies in human volunteers.

Development of an animal model for human NoV gastroenteritis might be based on infection of animals with human NoVs or the use of cultivable animal CVs as surrogates. Attempts to experimentally induce human NoV infection in NHP species such as common marmosets, cotton top tamarins, cynomolgus macaques, pigtail macaques, rhesus macaques and chimpanzees almost invariably resulted in asymptomatic infections [8], [10], [12], [31]. In one study however, a symptomatic infection characterized by diarrhea and vomiting of newborn pigtail macaques was induced following inoculation with the Toronto virus [12], but these results have not been confirmed by other studies. Moreover, detection of CVs or CV antibodies, including NoVs was reported in colonies with captive NHPs [27], [29], [30], [36]. In captive NHP colonies, CVs have been detected in stool samples of animals with symptoms of diarrhea. Recent data suggest an ∼80% ReCV seroprevalence in rhesus macaques housed at three separate U.S. colonies [27, Sestak and Farkas, unpublished]. While ReCV seroprevalence rates are high in juvenile and adult rhesus monkeys, other NHP species exhibit different and/or lower seroprevalence rates. As suggested, these differences could be due to species-specific susceptibility/resistance factors such as HBGAs [19]. Considering the high TV seroprevalence combined with high rates of juvenile diarrhea-associated morbidity in these colonies, it is suspected that ReCV infections play an important role in the etiology of NHP gastroenteritis. Here we examined for the first time whether experimental challenge of juvenile rhesus macaques with the tissue culture-adapted prototype ReCV (TV) induces clinical disease. Although no equivalent inoculum of tissue culture-adapted human NoV was available, two additional animals were challenged with human NoV stool suspensions to compare the clinical outcome between TV- and human NoV-inoculated animals. In order to increase the chance of infection, both animals were inoculated with a mixture of GII.2 and GII.4 NoVs. Mixed NoV infections have been described in oyster-associated human NoV outbreaks [37]. Also, the existence of recombinant NoVs [38] indicates that mixed NoV infections do frequently take place.

Two out of three TV-inoculated macaques developed symptoms of gastroenteritis (diarrhea, fever and virus shedding in stools but no vomiting or reduced appetite were noticed) while the third remained asymptomatic. All three animals shed virus for more than a week (8–10 PID) even though maximum virus shedding reached ∼10^5^ viral copies per gram of stool only in HC55 and HB61. Quantitative evaluation of virus shed in these two macaques combined with the daily output of stools (Table 2) indicate that the amount of virus shed exceeded the amount of virus in the inoculum. Vomiting was not detected as a clinical sign of TV infection. This could be due to small number of animals in this study or other reasons. Studies with NoV-infected children suggest that ∼25–30% of these children do not vomit [39], [40]. In addition, still unknown differences between ReCV and NoV pathogenesis might also be linked with the lack of vomiting in TV-infected macaques.

All three TV-inoculated macaques developed ≥4 fold TV-specific VN antibody responses within 7 days PI, indicating an early memory response, consistent with low levels of VN antibodies prior to inoculation. VN antibodies reached their peak by PID 7–9 in all of the three animals and persisted at high levels up to 4–5 weeks PI. After 5 weeks PI, VN antibody titers started to decline, possibly due to absence of re-exposure(s) that take place in colony animals [14], [18]. It is also possible that the binding (ELISA) antibodies would be detected for much longer period of time than functional (VN) antibodies. Finally, it is important to highlight that the low levels (1∶40–1∶80) of VN antibodies were still present at the end of the study in TV-inoculated macaques. Consistent with these results, declining NoV immunity has also been observed in human volunteers [41], [42]. Thus, questions still remain regarding the longevity, magnitude, class and role of CV antibodies in protection.

Interestingly, VN antibody responses could not be detected in any of the TV-infected animals against FT285 a GI.2 ReCV isolate, indicating that the two strains likely belong to different serotypes. TV and FT285 ORF2 and capsid proteins share 69% nt and 74% aa homology (Farkas and Sestak unpublished), which is comparable with differences between different human NoV genotypes. If humoral immunity plays a role in protection against enteric CV infections, our results are translational for NoV vaccine design since multiple genetic types have been described for both ReCVs and human NoVs. At present, ∼10 cell culture-adapted ReCVs representing at least three genotypes are available in our laboratory. Consistent with previous reports, none of the two human NoVs used in this study caused clinical infection. Since all macaques secreted type-B HBGA in their saliva (not shown), and both NoV challenge strains were obtained from a type-B patient, it was predicted that both strains would replicate in macaques. However, only GII.2 virus was detected in macaque GI96, while both Gll.2 and Gll.4 viruses were shed by HB11 (Table 2). Although duration of shedding in HB11 was suggestive of virus replication, no antibody responses were detected against Minerva (GII.4) VLPs. Macaque GI96 had relatively high level (1∶800) of ELISA antibodies at the beginning of the study. This could explain the complete absence of GII.4 virus shedding in GI96. GII.2 VLPs were not available to evaluate the GII.2-specific seroresponses in this study. Low viral loads in stools of Norwalk virus-challenged rhesus macaques were also reported by Rockx and colleagues [10]. In their study, two animals stopped shedding virus by PID 2 while one animal shed up to PID 19 while it developed strong antibody responses. In the same study, marmosets and tamarins, but not cynomolgus macaques, shed the virus up to PID 4 but did not develop antibody responses. Taken together, these studies indicate that NHPs can be subclinically infected with human NoVs but the infection is very limited and its intensity varies in different species and individuals.

Histopathological examination of H&E-stained duodenum biopsies obtained at PID 3 demonstrated lymphocytic infiltration of the lamina propria in all five inoculated macaques while mild villous blunting was present in three TV-inoculated macaques. These signs were consistent to some degree with reported histopathology of NoV-infected humans [43]–[45]. Although no ultrastructural examination of enterocytes was performed by transmission electron microscopy, confocal microscopy examination of intestinal brush border and epithelial layer revealed no significant damage of enterocytes indicating the possibility that virus might have translocated into lamina propria without replicating in enterocytes. This finding differs from what was reported with human NoV-inoculated gnotobiotic pigs or calves that showed presence of viral particles inside the enterocytes by PID 3 [9], [46]. Notwithstanding, our results are consistent with reported findings of human NoV i.v.-inoculated chimpanzees which showed the presence of NoV antigens in intestinal lamina propria while exhibiting the signs of asymptomatic virus infection such as virus shedding in stools and seroconversion [31]. In all of these studies, enterocyte damage was minimal or undetectable regardless of the detection of virus antigen raising questions about CV pathogenesis and their exact host cell tropism. In case of TV-inoculated macaques, TV antigen-containing cells with perinuclear fluorescence were found in intestinal lamina propria (Figs. 4 and 5). In many of these cells TV-specific antibodies co-localized with calnexin, an endoplasmic reticulum marker, consistent with the formation of replication complexes reported for feline CV [47].

To determine the authenticity of TV-containing cells, several of the human-specific cell markers were utilized (Table 3). A few CD20+TV+ B lymphocytes were identified directly in duodenum lamina propria by confocal microscopy. Since suitability of intestinal pin-head-sized biopsies for quantitative cell phenotype analysis by confocal microscopy is limited, *in vitro* experiment with TV-inoculated PBMCs obtained from eight healthy macaques was performed, utilizing quantitative multicolor flow cytometry, to determine if CD20+TV+ B cells could be identified in these cultures (Figs. 6 and 7). The presence of CD20+TV+ B cells was demonstrated corroborating the confocal microscopy data. Most of the TV+ cells expressed HLA-DR antigen, suggesting a) involvement of TV+ lymphocytes in antigen presentation to other immune cells or b) possibility of activated lymphocytes being infected. Increased viral RNA load (p<0.05) that was detected in cultured PBMCs within 24 h following TV inoculation supported the flow cytometry data and was suggestive of virus replication. In study by Lay and colleagues, Norwalk virus failed to replicate in macrophages or dendritic cells derived from human peripheral blood [48]. A more recent study by Chan and colleagues indicates however that human NoV binds to the lamina propria and Brunner's gland cells of the human duodenum [49]. We have also incorporated CD123 marker to determine whether some B cells start to acquire dendritic cell (DC)-like features following their *in vitro* exposure to virus. Earlier reports by Bjorck and colleagues (1998) showed that CD19+ B cells can differentiate into DCs following cytokine stimulation [50]. Although commonly used as a DC marker, CD11c is also expressed by activated or leukemic B cells [51], [52]. Recently, a subpopulation of memory B cells was shown to express CD11c [53]. In our study we observed increased CD11c expression by TV-infected B cells suggesting the activation of these cells following TV infection.

**Table 3:** Antibodies used for immunofluorescent staining of duodenum biopsy tissues.

Antigen	Cell Type/Component	Isotype	Working	Manufacturer	Catalog Number
			Dilution		or Clone
TV calicivirus	TV-Infected (primary ab)	Rhesus Plasma	1∶50	TNPRC^a^	NA^b^
Rhesus IgG-FITC^c^	TV-Infected (secondary ab)	Goat IgGγ	1∶500	RDI-Fitzgerald	RDI-617102012
Human Calnexin	Endoplasmic Reticulum	Rabbit IgG	1∶100	Santa Cruz	SC-11397
Human Cytokeratin	Epithelial Cell	Mouse IgG1	1∶50	Biocare Medical	CKLMW 8/18
Human Villin	Intestinal Brush Border	Rabbit IgG	1∶3	Cell Signalling Tech.	2369
Human CD3	T-lymphocyte	Rabbit IgG	1∶100	Biocare Medical	CP215C
Human CD20	B-lymphocyte	Mouse IgG2a	1∶400	Dako	M0755
Human IBA-1	Macrophage	Mouse IgG1	1∶100	Santa Cruz	SC-32725
Human CD11c	Myeloid Dendritic Cell	Mouse IgG	1∶10	Biosource	3.9
TUNEL (assay)	Apoptotic Cell	NA	NA	Millipore	S7165
Human TG2	Tissue Transglutaminase 2^d^	Mouse IgG1	1∶100	ThermoScientific	MS-300-P1ABX
Nuclear DNA	Nucleus	NA	1∶1,000	Invitrogen	T-3605

a Tulane National Primate Research Center,^ b^not applicable,^ c^Fluorescein-Iso-Thio-Cyanate, ^d^Digestive enzyme.

The fact that TV was identified in both B and T cells is to some degree different from findings reported for murine NoVs that replicate efficiently in macrophage and dendritic cell lines [13], [25], [26]. Since Rhesus CD14+ cells can differentiate into dendritic cells *in vitro*, it will be interesting to evaluate if these cells will become permissive to TV infection. In summary, our results are consistent with findings of human NoV -infected chimpanzees that remained asymptomatic but showed the presence of NoV antigen in intestinal lamina propria [31]. Many features of CV infection in primates such as HBGA-associated genetic susceptibility to infection remain to be elucidated. In this proof-of-concept study, we demonstrated the potential of rhesus macaques using ReCV as a diarrheal disease model for human NoV. In future studies candidate animals will be selected by prescreening for the specific HBGAs and/or by using newborn SPF or animals completely naïve of previous ReCV exposure while using highly cytopathic ReCV strain for challenge.

## Materials and Methods

### Ethics statement

Approval for all veterinary procedures in this study had been obtained from the Tulane University Animal Care and Use Committee, Animal Welfare Assurance A-4499-01. Animals in this project were under the full care of veterinarians with the standards incorporated in the Guide to the Care and Use of Laboratory Animals (NIH) 78-23 (Revised, 1996). All veterinary procedures were performed only with sedated animals. Animal welfare and steps were taken to ameliorate suffering in accordance with the recommendations of the Weatherall report. The findings and conclusions in this article are those of the authors and do not necessarily represent the views of the Centers for Disease Control and Prevention. This article did receive clearance through the appropriate channels at the CDC prior to submission.

**Table 4:** Antibodies used for flow cytometry of *in vitro* cultured PBMCs.

Antigen	Cell Type/ Component	Isotype	Working	Manufacturer	Catalog Number
			Dilution		and/or Clone
TV calicivirus	TV-Infected (primary ab)	Rhesus Plasma	1:50	TNPRC^a^	NA^b^
Rhesus IgG	TV-Infected (secondary ab)	Goat IgGγ	1:100	RDI-Fitzgerald	RDI-617102012
Human CD3	T-lymphocyte	Mouse IgG1	1:32	BD Pharmingen	558124, SP34-2
Human CD20	B-lymphocyte	Mouse IgG2b	1:32	eBioscience	48-0209-42, 2H7
Human CD123	Plasmacytoid Dendritic Cell	Mouse IgG2a	1:8	BD Biosciences	554529, 7G3
Human CD11c	Myeloid Dendritic Cell	Mouse IgG2b	1:32	BD Biosciences	340714, S-HCL-3
Human HLA-DR	Antigen-Presenting Cell	Mouse IgG2a	1:32	BioLegend	307625, L243

a Tulane National Primate Research Center,^ b^not applicable.

### Animals, procedures and samples collected

Forty juvenile (<4 years-old) candidate rhesus macaques were identified from a group of 300 based on absence of TV-neutralizing serum antibodies [27]. Due to unrelated reasons, selected candidate animals were assigned for the study not immediately but after a delay, which resulted in low, marginal but detectable TV seroconversion. Five animals (1∶10–1∶40 VN antibodies) with no detectable virus shedding in feces were included in this study (Table 1). Only simian retrovirus- (SIV, SR and STLV) and enteric pathogen-free animals were selected using the diagnostic techniques described elsewhere [54], [55]. All five macaques were inoculated intragastrically and were housed under biosafety level two (BSL2) conditions. Three macaques were inoculated with tissue culture-adapted TV and two macaques with bacteria-free stool suspensions containing a mixture of GII.2 an GII.4 NoVs (Table 1). Human NoV positive stool samples (NF2002 and NF2003; GenBank accession numbers: JQ320072 and JQ320073) were collected during two family outbreaks occurring in May 2002 and March 2003 in Cincinnati, OH, respectively. The inoculum was prepared from 20% (w/v) stool suspensions that were clarified by low-speed centrifugation and filtered using 0.22 μm filters. Aliquots were stored at −80°C. Upon inoculation, body temperature and stool consistency were recorded daily up to post-inoculation day (PID) 40. Stool samples were collected each morning at indicated time points (Fig. 1) from the individual cage pans that were replaced daily. Diarrhea was defined above the score of 1.0 according to following key: (0 = normal/dry, 0.5 = normal, 1.0 = normal/soft, 1.5 = pasty, 2.0 = semiliquid, 2.5 = watery), while fever was defined above the 102.5 F: (<102.5 = normal, 102.5 = borderline, >102.5 = fever).

Samples of blood and plasma were obtained weekly and/or at indicated time points (Fig. 2) up to PID 80. Based on similar experiments with gnotobiotic piglets [14], pediatric endoscopy method was used at PID 3 to obtain ten pin-head-sized biopsies from the distal duodenum of all five inoculated and two negative control healthy macaques [56].

### Virus antibody assays

Plasma samples collected from all five macaques were tested for the presence of virus-specific antibodies. TV-inoculated macaques (HC55, HI77 and HB61) were tested for the presence and magnitude of TV virus-neutralizing (VN) antibodies [27]. Briefly, serum samples were titrated in duplicate wells of 96-well tissue culture plates (Corning Life Sciences, Lowell, MA) to neutralize 100 TCID_50_ TV (GI.1) or FT285 (GI.2) ReCVs. To avoid non-specific neutralization by complement, serum samples were heat inactivated. Virus/serum mix was incubated at 37°C for 1 h and transferred into wells seeded with 1×10^4^ LLC-MK2 cells/well in duplicate one day prior. Plates were stained with crystal violet at 72 h post infection (Sigma-Aldrich, St. Louis, MO). At this time point, all cells in the virus control exhibited CPE characterized by cellular degeneration and detachment. VN antibody titer was determined as the highest dilution of the serum sample at which the cell monolayer was at least 50% intact in duplicate wells.

Human NoV-inoculated macaques (HB11 and GI96) were tested for the presence of NoV-binding (ELISA) antibodies using human NoV GII.4 (Minerva) virus-like particles (VLPs), as described previously [27]. Briefly, VLPs diluted in PBS were coated onto 96-well microtiter plates (Dynex, Immulon, Dynatech) at 50 ng/well, overnight, at 4°C. Every second row of the 96-well plate was left uncoated and served as background control. Plates were blocked with 10% Blotto-PBS for 1 h at 37°C. Serum samples, two-fold diluted in 1% Blotto-PBS, starting from 1∶50 were added to two wells each, and the plates were incubated for 1 h at 37°C. Horseradish peroxidase (HRP)-conjugated goat anti-rhesus IgG (H+L) (Southern Biotech, Birmingham, AL) was added at 1∶3,500 dilution in 1% Blotto-PBS, and the plates were incubated for 1 h at 37°C. Following each step, the plates were washed five times with PBS-0.05% Tween 20 in an automated plate washer (ELx 405 Auto Plate Washer, Bio-tek Instruments Inc., Winooski, VT). Finally, TMB substrate (BD Biosciences, San Jose, CA) was added, the plates were incubated for 10 min at room temperature and the color development was stopped by 1 M H_3_PO_4_. Optical density (OD) values were recorded by the use of Tecan Spectra II microtiter plate reader (Tecan AG, Switzerland) at 450 nm. OD readings for each serum sample/dilution were determined as the average of difference between values obtained in the two VLP-coated and two uncoated wells. The cut-off was determined as the mean plus two standard deviations of the OD readings of the control (uncoated) wells.

### Histopathology, immunohistochemistry and confocal microscopy

Duodenum biopsies collected at PID 3 were used for histopathological evaluation of tissue architecture following the standard H&E staining method [56]. In addition, antibodies specific to primate T and B lymphocytes, epithelial cells, macrophages, myeloid dendritic cells, apoptotic cells and endoplasmic reticulum protein calnexin (Table 3) were used in conjunction with TV-specific antibodies (rhesus serum of ≥1∶1,280 VN antibody titer), to identify the potential TV-infected cells according to described immunohistochemistry protocols [56], [57].

### TV infection of peripheral blood mononuclear cells (PBMC) with TV

PBMCs were isolated from whole blood of 8 healthy, normal rhesus macaques by density gradient centrifugation using Lymphocyte Separation Medium (MP Biomedicals, LLC, Santa Ana, CA) and seeded in 24-well cell culture plates coated with collagen (BD Biosciences, San Diego, CA) at a concentration of 2×10^6^ cells per well. Cells were cultured at 37°C, 5% CO_2_ in RPMI 1640 medium containing 2 mM glutamine, 1 mM sodium pyruvate, 100 U/ml penicillin, and 100 mg/ml streptomycin (Mediatech, Inc., Manassas, VA) supplemented with 10% heat-inactivated FBS (Hyclone, Thermo Scientific, Waltham, MA). Immediately after seeding the cells, half of the wells were inoculated with tissue culture-adapted TV (10^4^ RNA copies per well). Cells were incubated with TV for up to 6 days before being harvested for flow cytometry and/or TV-qPCR.

### Quantitative ReCV detection

For the detection of TV in fecal specimens or plasma, viral RNA was isolated from clarified 10% fecal suspensions in PBS or 100 μl of plasma using the QIAamp® Viral RNA Mini kit according to manufacturer's instructions (Qiagen GmbH, Hilden, Germany). For TV detection in cultured PBMCs, cells plus media were processed at 2 h, 24 h and 6 days PI with TV. The samples corresponding to individual wells of 24-well cell culture plate were pelleted with high-speed centrifugation (14,000 rpm) and the pellet stored in RNALater (Ambion, Austin, TX) at −20°C. The PBMCs were pelleted and washed in RNALater solution one more time before being used for RNA extraction. Total RNA was extracted using the RNeasy Plus Micro kit (Qiagen GmbH, Hilden, Germany). Precipitated RNA from both fecal specimens and PBMC cultures was eluted in 28 µl of nuclease-free water (Integrated DNA Technologies, Coralville, IA), and 8 µl of the sample was used as the template in the RT-PCR. RNA, primed with both oligo(dT) and random hexamers, was reverse-transcribed into cDNA in 20 µl reaction mixtures using Superscript™ III First-Strand Synthesis SuperMix for RT-qPCR (Invitrogen, Carlsbad, CA) according to the manufacturer's protocol. For quantitative real-time PCR, 10 µl of the cDNA product was used as the template in 50 µl reaction mixtures using Platinum Quantitative PCR SuperMix-UDG with ROX (Invitrogen) according to the manufacturer's protocol. The TV-specific primer pairs and oligonucleotide probe used were as follows: FW 5′-GAGATTGGTGTCAAAACACTCTTTG (nt position 3,645–3,669 on the TV genome, EU391643), RV 5′-ATCCAGTGGCACACACAATTT (nt position 3,820–3,800) and 6-FAM-AGTTGATTGACCTGCTGTGTCA-BHQ (nt position 3,697–3,719). PCR reactions were run in duplicate on an ABI 7900HT Fast Real-Time PCR System (Applied Biosystems, Foster City, CA) using the following amplification conditions: 2 min at 50°C, 10 min at 95°C, 40 cycles of 15 sec at 95°C, 1 min at 60°C. The threshold for detection of fluorescence above background was set at 0.185 within the exponential phase of the amplification curve. A standard curve was generated for each assay using 10-fold dilutions (from 10^1^ to 10^7^ copies) of a linearized plasmid containing the full TV genome. The detection limit of the assay was 10–100 copies per reaction. Negative control samples were run alongside each RNA sample from mock-infected cultures.

### Human NoV detection by quantitative RT-PCR

For the detection of GII.2 and GII.4 human NoVs, viral RNA was extracted from clarified 10% fecal suspensions in PBS with the MagMax^TM^ -96 Viral RNA Isolation Kit (Ambion, Foster City, CA) on an automated KingFisher magnetic particle processor (Thermo Fisher Scientific, Pittsburg, PA) according to the manufacturer's instructions and eluted into 100 µl. Viral RNA was tested in a GI/GII duplex format using an AgPath-ID™ One-Step RT-PCR Kit for Probes (Applied Biosystems, Foster City, CA) on an 7,500 Realtime PCR platform (Applied Biosystems). The reaction mix of 25 µl consisted of 400 nM of each oligonucleotide primer Cog1F, Cog1R, Cog2F, and Cog2R and 200 nM of each TaqMan® Probe Ring 1C and Ring 2 [58]. Cycling conditions included reverse transcription for 10 min at 45°C, denaturation for 10 min at 95°C followed by 45 cycles of 15 sec at 95°C and 1 min at 60°C.

### Nested RT-PCR for human NoV detection

Since mixtures of GII.2 and GII.4 human NoVs were used to inoculate the HB11 and GI96 macaques and RT-PCR assay was not able to differentiate between the two strains, a nested RT-PCR was designed to evaluate the strain-specific shedding in collected stool samples. Briefly, viral RNA was reverse transcribed and amplified in 20 cycles by generic primers P289/P290 in a 25 μl reaction (AccessQuick RT-PCR system, Promega, Madison, WI). One μl of the RT-PCR reaction was used as template in a nested PCR and amplified with primers 62F/63R (GTGTTGGCAGCAGCTCTAGAAATC and GTAACTTCAGAGAGCGCACAGAGA, specific for NF2003) and 64F/65R (TCATCAGAACCTCATCTGGCCCAA and AAAGGAGAACAGGGAGTTTGCCTGG, specific for NF2002) in 25 μl reactions in 35 cycles. Reactions yielded a 215 and a 225 bp product, respectively, which could be differentiated on a 2% ethidium bromide-stained agarose gel.

### Flow cytometry

Cell surface marker and intracellular staining were performed as follows. PBMC cultures were harvested, low-speed centrifuged (1,300 rpm), and cell pellets were washed twice with PBS (pH 7.4) containing 0.1% sodium azide and 0.2% FBS. Fluorochrome-conjugated antibodies specific to human and/or rhesus macaque surface molecules (Table 4) as well the rhesus serum used as the primary antibody against TV, were added to cell suspensions and incubated in the dark for 30 min at RT. The cells were then washed twice with 1x Perm/Wash Buffer (BD Biosciences) and resuspended in 50 µl Perm/Wash Buffer. The permeabilized cells were stained with the primary antibody against TV for 30 min at 4°C, washed with Perm/Wash Buffer, and stained with the fluorochrome-conjugated secondary antibody (rhesus IgG-FITC, #617102012, RDI-Fitzgerald, Acton, MA) for 1 h, at 4°C. Following staining, cells were washed twice and resuspended in 300 µl of 1x BD Stabilizing Fixative (BD Biosciences). Stained and fixed cells were stored in 12×75 mm polystyrene round-bottom tubes (Becton Dickinson, Franklin Lakes, NJ) in the dark at 4°C for up to 48 h until analyzed by flow cytometry. Data were collected using the BD LSRII flow cytometer and analyzed using FACSDiva (BD Biosciences) and FlowJo software (Tree Star, Ashland, OR). A minimum of 50,000 events were collected per sample. Gating was set based on the CD3^+^ T lymphocyte and CD20^+^ B lymphocyte populations (Fig. 6A). The HLA-DR^+^, CD11c^+^, and CD123^+^ cell subsets within CD20^+^ B cell population were evaluated for the presence of TV (Fig. 7).

### Statistical analysis

Statistically significant differences between proportions of TV^+^, HLA-DR^+^, CD11c^+^, and CD123^+^ cells within CD3^+^ and CD20^+^ populations of control versus TV-inoculated PBMC cultures were determined by the Student's t-test assuming equal variances. A p value of <0.05 was considered significant. Statistically significant differences between TV loads in cultured PBMCs at different time points after inoculation were also determined using the Student's t-test, with a significance level of p<0.05.

## Supporting Information

Figure S1 **Duodenal biopsy from TV-negative macaque (A) and from TV-inoculated HC55 animal (B).** TV antibody-specific staining shows an intense, cytoplasmic fluorescence in biopsy tissue collected at PID 3 from TV-inoculated but not from negative control animal.
(TIFF)

Figure S2 **Individual channels (corresponding to calnexin and TV immunofluorescent staining) control data.** DIC  =  differential interference contrast.
(TIFF)

## References

[1] Farkas T, Sestak K, Wei C, Jiang X (2008). Characterization of a rhesus monkey calicivirus representing a new genus of Caliciviridae.. J Virol.

[2] L'Homme Y, Sansregret R, Plante-Fortier E, Lamontagne AM, Ouardani M (2009). Genomic characterization of swine caliciviruses representing a new genus of Caliciviridae.. Virus Genes.

[3] Wolf S, Reetz J, Otto P (2011). Genetic characterization of a novel calicivirus from a chicken.. Arch Virol.

[4] Patel MM, Hall AJ, Vinjé J, Parashar UD (2009). Noroviruses: A comprehensive review.. J Clin Virol.

[5] Scallan E, Hoekstra RM, Angulo FJ, Tauxe RV, Widdowson MA (2011). Foodborne illness acquired in the United States – major pathogens.. Emerg Infect Dis.

[6] Atmar RL (2010). Noroviruses – State of the Art.. Food Environ Virol.

[7] Zheng DP, Ando T, Fankhauser RL, Beard RS, Glass RI (2006). Norovirus classification and proposed strain nomenclature.. Virology.

[8] Wyatt RG, Greenberg HB, Dalgard DW, Allen WP, Sly DL (1978). Experimental infection of chimpanzees with the Norwalk agent of epidemic viral gastroenteritis.. J Med Virol.

[9] Cheetham S, Souza M, Meulia T, Grimes S, Han MG (2006). Pathogenesis of a genogroup II human norovirus in gnotobiotic pigs.. J Virol.

[10] Rockx BH, Bogers WM, Heeney JL, van Amerongen G, Koopmans MP (2005). Experimental norovirus infections in non-human primates.. J Med Virol.

[11] Souza M, Azevedo MS, Jung K, Cheetham S, Saif LJ (2008). Pathogenesis and immune responses in gnotobiotic calves after infection with the genogroup II.4-HS66 strain of human norovirus.. J Virol.

[12] Subekti DS, Tjaniadi P, Lesmana M, McArdle J, Iskandriati D (2002). Experimental infection of Macaca nemestrina with a Toronto Norwalk-like virus of epidemic viral gastroenteritis.. J Med Virol.

[13] Wobus CE, Karst SM, Thackray LB, Chang KO, Sosnovtsev SV (2004). Replication of Norovirus in cell culture reveals a tropism for dendritic cells and macrophages.. PLoS Biol.

[14] Cannon JL, Lindesmith LC, Donaldson EF, Saxe L, Baric RS (2009). Herd immunity to GII.4 noroviruses is supported by outbreak patient sera.. J Virol.

[15] Lindesmith L, Moe C, Lependu J, Frelinger JA, Treanor J (2005). Cellular and humoral immunity following Snow Mountain virus challenge.. J Virol.

[16] Hutson AM, Altmar RL, Graham DY, Estes MK (2002). Norwalk virus infection and disease is associated with ABO histo-blood group type.. J Infect Dis.

[17] Lindesmith L, Moe C, Marionneau S, Ruvoen N, Jiang X (2003). Human susceptibility and resistance to Norwalk virus infection.. Nat Med.

[18] Tan M, Jiang X (2010). Norovirus gastroenteritis, carbohydrate receptors, and animal models.. PLoS Pathog.

[19] Farkas T, Cross RW, Hargitt E, Lerche NW, Morrow AL (2010). Genetic diversity and histo-blood group antigen interactions of rhesus enteric caliciviruses.. J Virol.

[20] Barron EL, Sosnovtsev SV, Bok K, Prikhodko V, Sandoval-Jaime C (2011). Diversity of murine norovirus strains isolated from asymptomatic mice of different genetic backgrounds within a single U.S. research institute.. PLoS ONE.

[21] Bok K, Prikhodko VG, Green KY, Sosnovtsev SV (2009). Apoptosis in murine norovirus-infected RAW264.7 cells is associated with downregulation of survivin.. J Virol.

[22] Gerondopoulos A, Jackson T, Monaghan P, Doyle N, Roberts LO (2010). Murine norovirus-1 cell entry is mediated through a non-clathrin-, non-caveolae-, dynamin- and cholesterol-dependent pathway.. J Gen Virol.

[23] LoBue AD, Thompson JM, Lindesmith L, Johnston RE, Baric RS (2009). Alphavirus-adjuvanted norovirus-like particle vaccines: heterologous, humoral, and mucosal immune responses protect against murine norovirus challenge.. J Virol.

[24] LoBue AD, Lindesmith LC, Baric RS (2010). Identification of cross-reactive norovirus CD4+ T cell epitopes.. J Virol.

[25] Perry JW, Wobus CE (2010). Endocytosis of murine norovirus 1 into murine macrophages is dependent on dynamin II and cholesterol.. J Virol.

[26] Taube S, Perry JW, Yetming K, Patel SP, Auble H (2009). Ganglioside-linked terminal sialic acid moieties on murine macrophages function as attachment receptors for murine noroviruses.. J Virol.

[27] Farkas T, Dufour J, Jiang X, Sestak K (2010). Detection of norovirus-, sapovirus- and rhesus enteric calicivirus-specific antibodies in captive juvenile macaques.. J Gen Virol.

[28] Wei C, Farkas T, Sestak K, Jiang X (2008). Recovery of infectious virus by transfection of in vitro-generated RNA from tulane calicivirus cDNA.. J Virol.

[29] Jiang B, McClure HM, Fankhauser RL, Monroe SS, Glass RI (2004). Prevalence of rotavirus and norovirus antibodies in non-human primates.. J Med Primatol.

[30] Wang Y, Tu X, Humphrey C, McClure H, Jiang X (2007). Detection of viral agents in fecal specimens of monkeys with diarrhea.. J Med Primatol.

[31] Bok K, Parra GI, Mitra T, Abente E, Shaver CK (2011). Chimpanzees as an animal model for human norovirus infection and vaccine development.. Proc Natl Acad Sci U S A.

[32] Patel MM, Widdowson MA, Glass RI, Akazawa K, Vinje J (2008). Systematic literature review of role of noroviruses in sporadic gastroenteritis.. Emerg Infect Dis.

[33] Kapikian AZ, Wyatt RG, Dolin R, Thornhill TS, Kalica AR (1972). Visualization by immune electron microscopy of a 27-nm particle associated with acute infectious nonbacterial gastroenteritis.. J Virol.

[34] El-Kamary SS, Pasetti MF, Mendelman PM, Frey SE, Bernstein DI (2010). Adjuvanted intranasal Norwalk virus-like particle vaccine elicits antibodies and antibody-secreting cells that express homing receptors for mucosal and peripheral lymphoid tissues.. J Infect Dis.

[35] Atmar RL, Bernstein DI, Harro CD, Al-Ibrahim MS, Chen WH (2011). Norovirus vaccine against experimental human Norwalk Virus illness.. N Engl J Med.

[36] Smith AW, Skilling DE, Anderson MP, Benirschke K (1985). Isolation of primate calicivirus Pan paniscus type 1 from a douc langur (Pygathrix nemaeus l.).. J Wildl Dis.

[37] Symes SJ, Gunesekere IC, Marshall JA, Wright PJ (2007). Norovirus mixed infections in an oyster-associated outbreak: an opportunity for recombination.. Arch Virol.

[38] Bull RA, Tanaka MM, White PA (2007). Norovirus recombination.. J Gen Virol.

[39] Kaplan JE, Gary GW, Baron RC, Singh N, Schonberger LB (1982). Epidemiology of Norwalk gastroenteritis and the role of Norwalk virus in outbreaks of acute nonbacterial gastroenteritis.. Ann Intern Med.

[40] Yang SY, Hwang KP, Wu FT, Wu HS, Hsiung CA (2010). Epidemiology and clinical peculiarities of norovirus and rotavirus infection in hospitalized young children with acute diarrhea in Taiwan, 2009.. J Microbiol Immunol Infect.

[41] Parrino TA, Schreiber DS, Trier JS, Kapikian AZ, Blacklow NR (1977). Clinical immunity in acute gastroenteritis caused by Norwalk agent.. N Engl J Med.

[42] Wyatt RG, Dolin R, Blacklow NR, DuPont HL, Buscho RF (1974). Comparison of three agents of acute infectious nonbacterial gastroenteritis by cross-challenge in volunteers.. J Infect Dis.

[43] Agus SG, Dolin R, Wyatt RG, Tousimis AJ, Northrup RS (1973). Acute infectious nonbacterial gastroenteritis: intestinal histopathology. Histologic and enzymatic alterations during illness produced by the Norwalk agent in man.. Ann Intern Med.

[44] Dolin R, Levy AG, Wyatt RG, Thornhill TS, Gardner JD (1975). Viral gastroenteritis induced by the Hawaii agent. Jejunal histopathology and serologic response.. Am J Med.

[45] Schreiber DS, Blacklow NR, Trier JS (1974). The small intestinal lesion induced by Hawaii agent acute infectious nonbacterial gastroenteritis.. J Infect Dis.

[46] Otto PH, Clarke IN, Lambden PR, Salim O, Reetz J (2011). Infection of calves with bovine norovirus GIII.1 strain Jena virus: an experimental model to study the pathogenesis of norovirus infection.. J Virol.

[47] Bailey D, Kaiser WJ, Hollinshead M, Moffat K, Chaundry Y (2010). Feline calicivirus p32, p39 and p30 proteins localize to the endoplasmic reticulum to initiate replication complex formation.. J Gen Virol.

[48] Lay MK, Atmar RL, Guix S, Bharadwaj U, He H (2010). Norwalk virus does not replicate in human macrophages or dendritic cells derived from the peripheral blood of susceptible humans.. Virology.

[49] Chan MC, Ho W, Sung JJ (2011). *In vitro* whole-virus binding of a norovirus genogroup II genotype 4 strain to cells of the lamina propria and Brunner's glands in the human duodenum.. J Virol.

[50] Bjorck P, Kincade PW (1998). CD19+ pro-B cells can give rise to dendritic cells in vitro.. J Immunol.

[51] Molica S, Datillo A, Mannella A, Levato D (1994). CD11c expression in B-cell chronic lymphocytic leukemia. A comparison of results obtained with different monoclonal antibodies.. Haematologica.

[52] Postigo AA, Corbi AL, Sanchez-Madrid F, de Landazuri MO (1991). Regulated expression and function of CD11c/CD18 integrin on human B lymphocytes. Relation between attachment to fibrinogen and triggering of proliferation through CD11c/CD18.. J Exp Med.

[53] Ehrhardt GR, Hijikata A, Kitamura H, Ohara O, Wang JY (2008). Discriminating gene expression profiles of memory B cell subpopulations.. J Exp Med.

[54] Sestak K, Merritt CK, Borda J, Saylor E, Schwamberger SR (2003). Infectious agent and immune response characteristics of chronic enterocolitis in captive rhesus macaques.. Infect Immun.

[55] Sestak K, McNeal MM, Choi A, Cole MJ, Ramesh G (2004). Defining T-cell-mediated immune responses in rotavirus-infected juvenile rhesus macaques.. J Virol.

[56] Mazumdar K, Alvarez X, Borda JT, Dufour J, Martin E (2010). Visualization of transepithelial passage of the immunogenic 33-residue peptide from alpha-2 gliadin in gluten-sensitive macaques.. PLoS One.

[57] Ramesh G, Alvarez X, Borda JT, Aye PP, Lackner AA (2005). Visualizing cytokine-secreting cells in situ in the rhesus macaque model of chronic gut inflammation.. Clin Diagn Lab Immunol.

[58] Vega E, Barclay L, Gregoricus N, Williams K, Lee D (2011). Novel surveillance network for norovirus gastroenteritis outbreaks, United States.. Emerg Infect Dis.

