# Supplementary figures and images for: Experimental Inoculation of Juvenile Rhesus Macaques with Primate Enteric Caliciviruses
Source: PLoS One. 2012 May 30;7(5):e37973. doi: 10.1371/journal.pone.0037973 (PMC3364207; doi:10.1371/journal.pone.0037973)

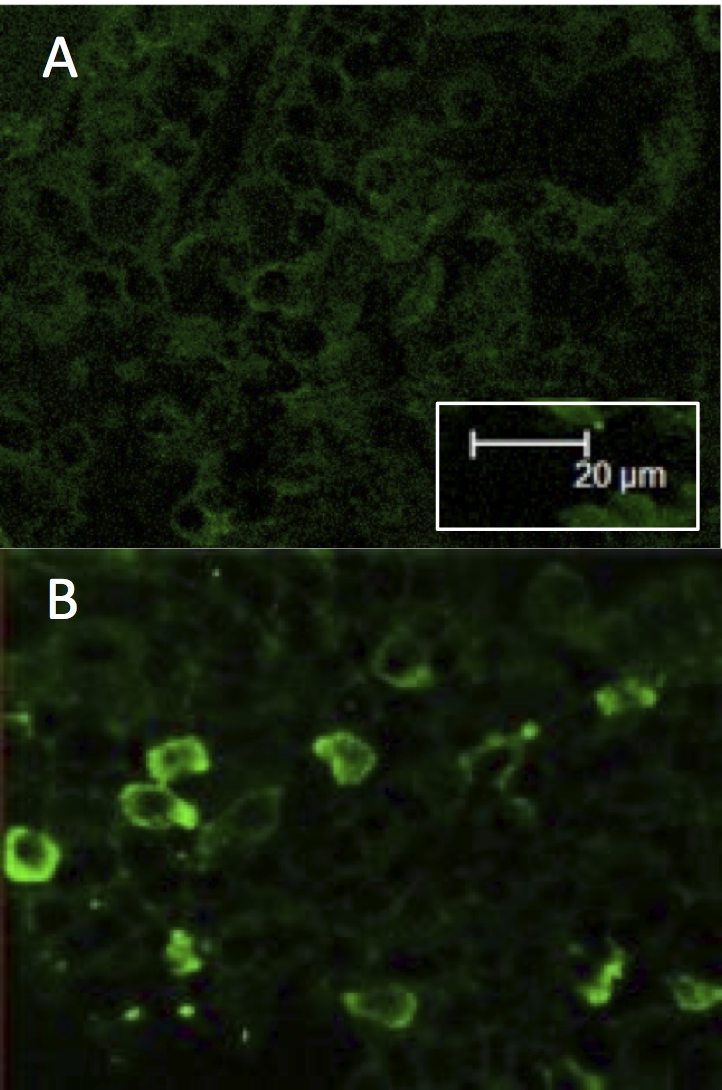

Supplement: Figure S1 — Duodenal biopsy from TV-negative macaque (A) and from TV-inoculated HC55 animal (B). TV antibody-specific staining shows an intense, cytoplasmic fluorescence in biopsy tissue collected at PID 3 from TV-inoculated but not from negative control animal. (TIFF) [file pone.0037973.s001.tiff]

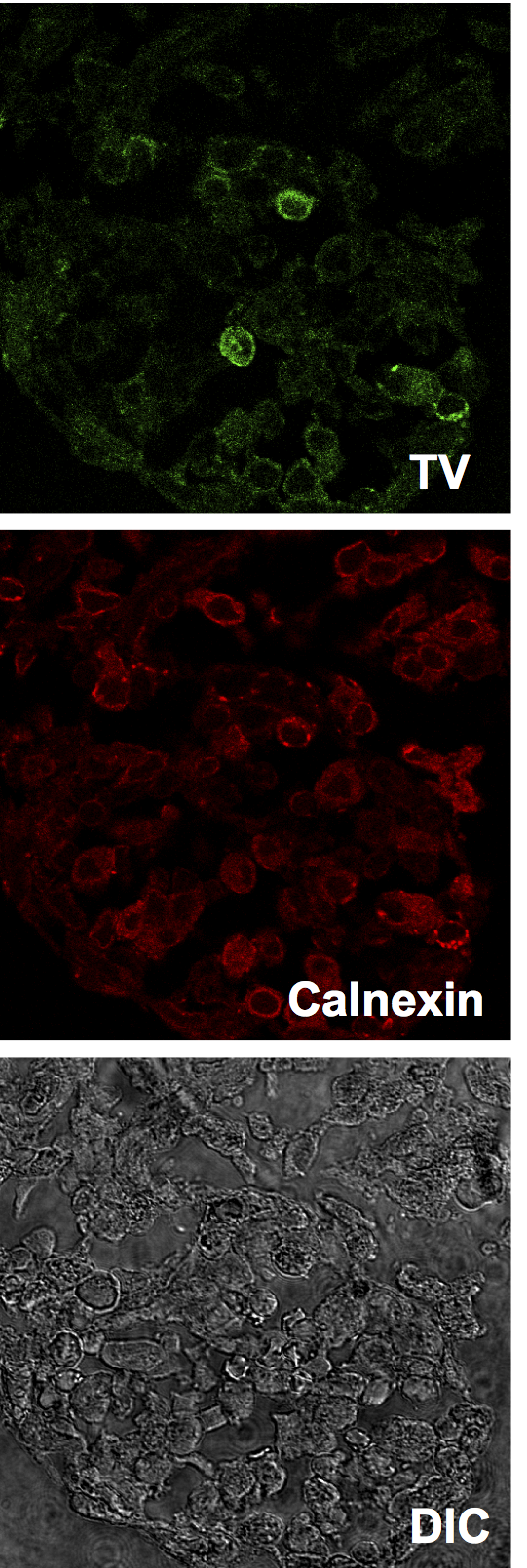

Supplement: Figure S2 — Individual channels (corresponding to calnexin and TV immunofluorescent staining) control data. DIC = differential interference contrast. (TIFF) [file pone.0037973.s002.tiff]
